# ACE2 Interaction Networks in COVID-19: A Physiological Framework for Prediction of Outcome in Patients with Cardiovascular Risk Factors

**DOI:** 10.3390/jcm9113743

**Published:** 2020-11-21

**Authors:** Zofia Wicik, Ceren Eyileten, Daniel Jakubik, Sérgio N. Simões, David C. Martins, Rodrigo Pavão, Jolanta M. Siller-Matula, Marek Postula

**Affiliations:** 1 Centro de Matemática, Computação e Cognição, Universidade Federal do ABC, Santo Andre 09606-045, Brazil; zofiawicik@gmail.com (Z.W.); david.martins@ufabc.edu.br (D.C.M.J.); rpavao@gmail.com (R.P.); 2 Department of Experimental and Clinical Pharmacology, Medical University of Warsaw, Center for Preclinical Research and Technology CEPT, 02-091 Warsaw, Poland; ceyileten@wum.edu.pl (C.E.); djakubik@wum.edu.pl (D.J.); mpostula@wum.edu.pl (M.P.); 3 Federal Institute of Education, Science and Technology of Espírito Santo, Serra, Espírito Santo 29056-264, Brazil; sergio@ifes.edu.br; 4 Department of Internal Medicine II, Division of Cardiology, Medical University of Vienna,1090 Vienna, Austria

**Keywords:** ACE2, COVID-19, SARS-CoV-2, cardiovascular, gene expression, miRNA, therapeutic target, microRNA, miR

## Abstract

Background: Severe acute respiratory syndrome coronavirus 2 (SARS-CoV-2) infection (coronavirus disease 2019; COVID-19) is associated with adverse outcomes in patients with cardiovascular disease (CVD). The aim of the study was to characterize the interaction between SARS-CoV-2 and Angiotensin-Converting Enzyme 2 (ACE2) functional networks with a focus on CVD. Methods: Using the network medicine approach and publicly available datasets, we investigated ACE2 tissue expression and described ACE2 interaction networks that could be affected by SARS-CoV-2 infection in the heart, lungs and nervous system. We compared them with changes in ACE-2 networks following SARS-CoV-2 infection by analyzing public data of human-induced pluripotent stem cell-derived cardiomyocytes (hiPSC-CMs). This analysis was performed using the Network by Relative Importance (NERI) algorithm, which integrates protein-protein interaction with co-expression networks. We also performed miRNA-target predictions to identify which miRNAs regulate ACE2-related networks and could play a role in the COVID19 outcome. Finally, we performed enrichment analysis for identifying the main COVID-19 risk groups. Results: We found similar ACE2 expression confidence levels in respiratory and cardiovascular systems, supporting that heart tissue is a potential target of SARS-CoV-2. Analysis of ACE2 interaction networks in infected hiPSC-CMs identified multiple hub genes with corrupted signaling which can be responsible for cardiovascular symptoms. The most affected genes were EGFR (Epidermal Growth Factor Receptor), FN1 (Fibronectin 1), TP53, HSP90AA1, and APP (Amyloid Beta Precursor Protein), while the most affected interactions were associated with MAST2 and CALM1 (Calmodulin 1). Enrichment analysis revealed multiple diseases associated with the interaction networks of ACE2, especially cancerous diseases, obesity, hypertensive disease, Alzheimer’s disease, non-insulin-dependent diabetes mellitus, and congestive heart failure. Among affected ACE2-network components connected with the SARS-Cov-2 interactome, we identified AGT (Angiotensinogen), CAT (Catalase), DPP4 (Dipeptidyl Peptidase 4), CCL2 (C-C Motif Chemokine Ligand 2), TFRC (Transferrin Receptor) and CAV1 (Caveolin-1), associated with cardiovascular risk factors. We described for the first time miRNAs which were common regulators of ACE2 networks and virus-related proteins in all analyzed datasets. The top miRNAs regulating ACE2 networks were miR-27a-3p, miR-26b-5p, miR-10b-5p, miR-302c-5p, hsa-miR-587, hsa-miR-1305, hsa-miR-200b-3p, hsa-miR-124-3p, and hsa-miR-16-5p. Conclusion: Our study provides a complete mechanistic framework for investigating the ACE2 network which was validated by expression data. This framework predicted risk groups, including the established ones, thus providing reliable novel information regarding the complexity of signaling pathways affected by SARS-CoV-2. It also identified miRNAs that could be used in personalized diagnosis in COVID-19.

## 1. Introduction

At the end of 2019 in Wuhan (China), a novel coronavirus named severe acute respiratory syndrome coronavirus 2 (SARS-CoV-2) was discovered [1]. The clinical manifestations of SARS-CoV-2 infection, named coronavirus disease 2019 (COVID-19), vary in severity from asymptomatic infection to acute viral pneumonia with fatal outcome. Nearly half of patients who were at risk of the acute course of the disease suffered from comorbidities including hypertension, diabetes mellitus (DM), and coronary heart disease [2,3]. Importantly, COVID-19 is associated with an increased risk for mortality and adverse cardiovascular events among patients with underlying cardiovascular diseases (CVD) [4]. A similar association between the virus and CVD was observed during previous coronavirus outbreaks such as Middle-East respiratory syndrome coronavirus (MERS) or severe acute respiratory syndrome coronavirus (SARS-CoV) [5,6]. Therefore, these data suggest a common factor that is associated with the pathogenesis of COVID-19 and CVD. The etiology of cardiac injury in COVID-19, however, remains unclear. It is hypothesized that cardiac injury may be ischemia mediated, and the profound inflammatory and hemodynamic impacts seen in COVID-19 can cause atherosclerotic plaque rupture or oxygen supply-demand mismatch resulting in ischemia [7]. Most probably, the link between cardiovascular complications and infection may be related to angiotensin-converting enzyme 2 (ACE2), which was found to act as a functional receptor for SARS-CoV-2 [8].

ACE2 is a multi-action cell membrane enzyme that is widely expressed in the lungs, heart tissue, intestine, kidneys, central nervous system, testis, and liver [9]. During the 20 years since its discovery, investigations targeting the complex role of this enzyme have established ACE2 as an important regulator in hypertension, heart failure (HF), myocardial infarction (MI), DM, and lung diseases [10,11]. The viral entry to cells is determined by the interaction between the SARS-CoV-2 spike (S) protein and the N-terminal segment of ACE2 protein, with a subsequent decrease in ACE2 surface expression, which may be enhanced by cofactor transmembrane protease serine 2 (TMPRSS2) [12]. Publications of independent research groups have shown that cardiomyocytes can be infected by SARS-CoV-2. The virus can enter into human-induced pluripotent stem cell-derived cardiomyocyte (hiPSC-CMs) via ACE2, and the viral replication and cytopathic effects induce hiPSC-CM apoptosis and cessation of beating after 72 h of infection while inhibiting metabolic pathways and suppressing ACE2 expression during this initial infection stage [13]. Moreover, SARS-CoV-2 undergoes a full replication circle and induces a cytotoxic response in cardiomyocytes, by inducing pathways related to viral response and interferon signaling, apoptosis and reactive oxygen stress [14]. Consistently, ACE2 mRNA and protein are expressed in hiPSC-CM, whereas TMPRSS2 was detected only at very low levels by RNA sequencing [14]. Finally, SARS-CoV-2 was invariably detected in cardiomyocytes of COVID-19 patients without clinical signs of cardiac involvement, with degrees of injury ranging from the absence of cell death and subcellular alteration hallmarks to intracellular oedema and sarcomere ruptures [15]. These findings support that heart tissue can be infected by SARS-Cov-2.

As a consequence of SARS-CoV-2 infection, downregulated ACE2 pathways may lead to myocardial injury, fibrosis, and inflammation which may be responsible for adverse cardiac outcomes [16]. In line with these findings, several reports have linked SARS-CoV-2 infection with myocardial damage and HF, accompanied by acute respiratory distress syndrome (ARDS), acute kidney injury, arrhythmias, and coagulopathy. The incidence of myocardial injury ranged from 7 to 28% depending on the severity of COVID-19, accompanied by increased levels of cardiac troponins, creatinine kinase–myocardial band, myo-hemoglobin, and N-terminal pro-B-type natriuretic peptide (NT-proBNP) [17,18,19].

In the current work, we characterized the ACE2 interaction network in the context of myocardial injury. Our quantitative in silico analysis pointed out: (1) the potential tissues and organs which can be infected by SARS-CoV-2; (2) the top ACE2 interactors associated with the virus-related processes with altered co-expression networks in hiPSC-CMs after SARS-CoV-2 infection, which are likely to play a role in the development of CVD; (3) signaling pathways associated with alteration of ACE2 networks; (4) prediction of risk groups in COVID-19; (5) connections between ACE2 and SARS-Cov2 interactomes, as well as ACE2 co-expression networks in hiPSC-CMs; (6) the most promising microRNAs (miRNAs, miR) regulating ACE2 networks for potential diagnostic and prognostic applications.

Our comprehensive analysis investigating ACE2 receptor-related interaction networks, their connection with SARS-CoV-2 interactome, enriched signaling pathways, miRNAs and associated diseases provide precise targets for developing predictive tools, with the potential for reducing the health, personal and economic consequences of the pandemic.

## 2. Materials and Methods

### 2.1. Data Collection

ACE2-associated genes used for constructing interaction networks were extracted from the Kyoto Encyclopedia of Genes and Genomes (KEGG) pathway database (23 genes from renin-angiotensin system (RAS) pathway) [20]; Cytoscape stringApp-Search Tool for the Retrieval of Interacting Genes/Proteins (top 40 ACE2 interactors) [21], Archs4 database (https://amp.pharm.mssm.edu/archs4, top 20 genes with correlated expression), the GeneCards database (https://www.genecards.org, five interactors and four sister terms), and literature search [22,23,24]. In total, we collected 69 genes, which were used further for miRNA prediction analysis and constructing interaction networks. In all steps of data integration and bioinformatic analyses, we used our R package wizbionet [25].

### 2.2. Tissue Expression Analysis

The tissue expression of ACE2 and TMPRSS2 was evaluated based on a dataset downloaded from the TISSUES 2.0 database and the Genotype-Tissue Expression (GTEx) project database [26]. TISSUES 2.0 database integrates transcriptomics datasets from multiple sources and proteomics datasets from humans and other organisms, quantifying gene expression confidence scores across tissues. All tissues were sorted by the decreasing expression confidence of ACE2 and TMPRSS2. Additionally, mean expression confidence for the ACE2 network was calculated for each tissue from the TISSUES 2.0 database. Gene expression confidence scores from this database were also mapped on the visualization of the interaction networks.

### 2.3. Interaction Network Analysis

#### 2.3.1. ACE2 Interactome

To analyze connections between ACE2 and other genes, we constructed a Protein-Protein Interaction PPI network in Cytoscape 3.7.2, using human interactome data from the stringApp 1.5.1 database, including known and predicted protein-protein interactions [21,27]. Interaction networks were composed of a set of genes (nodes) connected by edges that represent functional relationships among these genes. As suggested by the StringApp, we took into account connections with edge interaction confidence cut-off > 0.4 (medium confidence), with 1 being the highest possible confidence and 0 the lowest. We compared the complete tissue-specific ACE2 network, across the heart, lungs, and nervous system, as well as the virus-infection-related proteins network. Selection criteria for the tissue-specific networks were gene expression confidence score > 2.

#### 2.3.2. NERI Method

NERI [28] is a method that computes the relative importance of genes related to seeds. The method is based on the Network Medicine Hypotheses: Locality, Disease Module, and Network Parsimony. It integrates the PPI network with expression data (from the two conditions, e.g., control and disease), and uses a previously chosen seed genes list. NERI computes two relative importance scores for each gene, one score for each expression condition (control and disease). This is done by selecting the best of the shortest paths (based on the Parsimony Hypothesis) from seeds to their neighborhood (based on Locality and Disease Module Hypotheses) and taking into account the expression condition. The adopted criterion to evaluate a path is the modified Kendall’s concordance (a way to measure a group correlation) of expression of genes in the given path—the more concordant a path is, the better. Then, the relative importance score of a given gene is the sum of all concordances of the selected paths to which the gene belongs, weighted by the proximity to the seeds. This procedure is executed for two conditions independently, generating two networks (e.g., control and disease networks). In the end, the NERI method performs the differential network analyses, which outputs two ranked lists of genes (X, Δ): one based on the sum of the relative importance scores, and another based on the normalized difference between the relative importance. The first one (X) prioritizes genes with party hub features, possessing high topological centrality and, at the same time, high co-expression relative to the seed genes. The second (Δ) prioritizes the most altered genes between two conditions as described before [28]. In the present study, we used the NERI algorithm to analyze raw expression signals obtained from the GSE150392 dataset from the Gene Expression Omnibus (GEO) database. Besides, we used the interactome data from The Biological General Repository for Interaction Datasets (BIOGRID) to construct the PPI network and, as seed genes, the 69 ACE2-related interactors collected as described in the method section. Differential expression analysis for visualization was performed using the Mann Whitney test with *p* False Discovery Rate (FDR) corrected < 0.05.

### 2.4. Extraction of Disease-Relevant Ontological Terms

To improve the interpretation of the gene functions, we mined the Gene Ontology (GO) database using the biomaRt R package for extracting GO terms and further genes associated with the following processes: “inflammation” (22 GO terms; 648 genes), “coagulation” (18 GO terms; 223 genes) [29], “angiogenesis” (24 GO terms; 535 genes), “cardiac muscle functions” (176 GO terms; 524 genes), “muscle hypertrophy” (16 GO terms; 85 genes), and “fibrosis” (23 GO terms; 263 genes). A similar methodology was used to extract genes potentially related to “viral infection” (120 GO terms, 1047 genes). Disease relevant gene lists were extracted from the http://t2diacod.igib.res.in/ database [30]. From this database, we used atherosclerosis, nephropathy, CVD, and neuropathy datasets. Diabetes-related genes were extracted from the StringApp disease database for the term “diabetes type-2”. GO term lists used for the gene extraction are shown in Table S1.

### 2.5. Enrichment Analysis

Enrichment analysis is a computational method for increasing the likelihood of identifying the most significant biological processes related to the study [31]. Enrichment analysis of the diseases and networks was performed with the EnrichR database [32], using Fisher’s exact with Benjamini and Hochberg correction, while the reference was the precomputed background for each term in each gene set library. Signaling pathways were analyzed using BioPlanet2019 and Human KEGG 2019 datasets. Diseases were analyzed using DisGenet Dataset and Diseases AutoRIF Gene Lists datasets. In all statistical analyses, the significance cutoff was set to corrected *p*-value ≤ 0.05.

### 2.6. miRNA Predictions

To identify miRNAs regulating ACE2 related genes, we used the R and multimiR package with default settings, similar to previous publications from our group [29,33,34,35]. Interaction networks between ACE2-related genes and miRNAs were constructed in R and exported to Cytoscape 3.7.2. Next, the interaction networks were merged with the predicted PPI network for ACE2 and the network constructed using StringApp. Both networks were merged using official gene symbols and Ensembl gene IDs.

## 3. Results

### 3.1. ACE2 Tissue-Specific Expression

**Figure 1:**
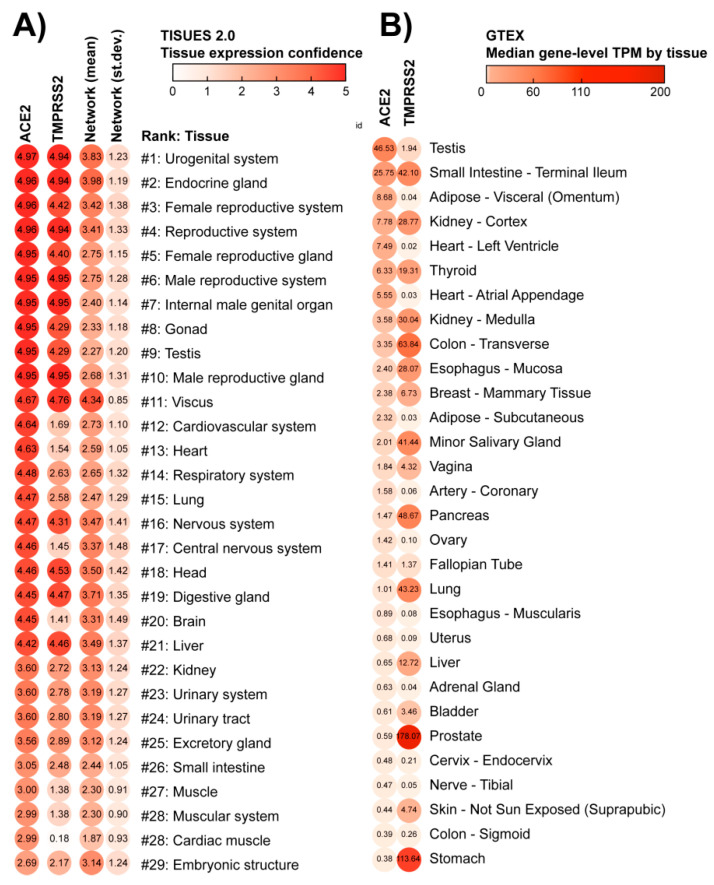
Tissues sorted by the potential of being infected by SARS-CoV-2. These lists of tissues were generated according to the concentration of membrane receptors Angiotensin-Converting Enzyme 2 (ACE2) and Transmembrane Protease Serine 2 (TMPRSS2), obtained from (**A**) TISSUES 2.0 database expression confidence values, and (**B**) Genotype-Tissue Expression (GTEx) project Transcripts Per Million (TPM) values. The virus starts the cell infection by binding to ACE2, a major hub in multiple physiological processes: this binding can block ACE2 network activity. However, the virus will enter the host cell when TMPRSS2 cleavages ACE2. The first column depicts the average gene and protein expression confidence for the ACE2 receptor; the second column depicts the average expression confidence of TMPRSS2. The mean and standard deviation of expression confidence across 69 genes/proteins of the ACE2 network are presented in the third and fourth columns of panel A, respectively. Notice that the lungs and respiratory system are ranked as #14–15 in the TISSUES 2.0 list, while the heart and cardiovascular system are #12–13. Nervous and reproductive systems are ranked as #16–17 and #1–10, respectively.

To evaluate the potential susceptibility of the heart for SARS-CoV-2 infection, we ranked all 6668 human tissues from the TISSUES 2.0 database, based on the provided gene expression confidence (scale 0–5). This analysis revealed that lungs and respiratory systems are in the 14th and 15th place in terms of ACE2 expression confidence, after heart and cardiovascular systems (12th and 13th place, respectively), and before the nervous system (16th place; Figure 1A). TMPRSS2 gene expression confidence was lower in the lungs and heart than in the nervous system. The mean expression of 69 genes from the network was highest in the nervous system in comparison to heart and respiratory system tissues. Exceptionally high scores for ACE2 expression were assigned to urogenital and reproductive tissues. As an additional validation, we used the expression dataset from the GTEx database, which confirmed our findings when using the TISSUES 2.0 database (Figure 1B). According to the GTEx database, the testis showed the highest expression of ACE2, while the prostate showed high expression of TMPRSS2. Heart-related tissues were located respectively in 5th, 7th, and 15th place. In turn, the gastrointestinal tract in GTEx appears in second place while in the TISSUES 2.0 database it was not present among the top hits. In both datasets, female reproductive glands (TISSUES 2.0) and mammary glands (GTEx) showed a high expression of the ACE2 receptor.

### 3.2. Construction of the Complete ACE2 Network

**Figure 2:**
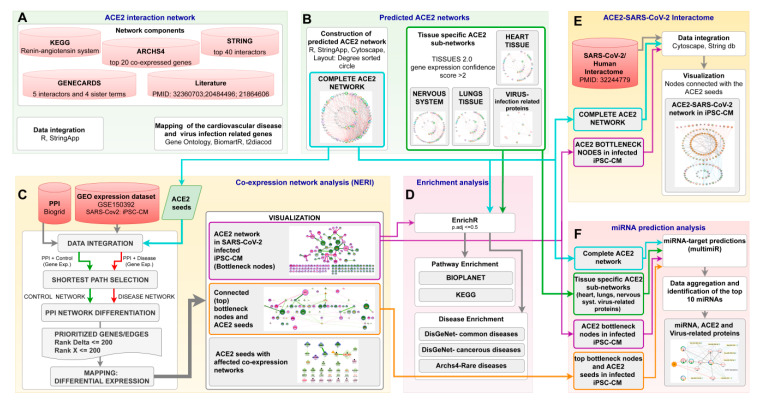
The workflow of bioinformatic analyses. (**A**) data collection to construct complete ACE2 (Angiotensin-Converting Enzyme 2) network; (**B**) generation of the complete ACE2 network as well as tissue-specific sub-networks; (**C**) ACE2-related co-expression network analysis of human-induced pluripotent stem cell-derived cardiomyocyte (hiPSC-CMs) 72 h post-infection with SARS-CoV2 using Network by Relative Importance (NERI) algorithm; (**D**) Enrichment analysis of signaling pathways and diseases related to alterations in ACE2 networks; (**E**) integration of complete ACE2 network with NERI; and (**F**) miRNA prediction analysis in ACE2 related networks.

By using the available literature and interactome data, we made an attempt to construct the ACE2 network as completely as possible based on the interactions with other genes and proteins. We developed the network based on three assumptions from the network medicine field: (i) disease module hypothesis: gene-products associated with the same disease phenotype tend to form a cluster in the PPI network; (ii) network parsimony: shortest paths between known disease genes often coincide with disease pathways; (iii) local hypothesis: gene products associated with similar diseases are likely to strongly interact with each other [36]. Besides protein–protein interactions, we also included genes that showed correlated expression with ACE2, taking into account that these genes could not yet have a strong representation in PPI databases. This synthesis aimed to gather available knowledge regarding the ACE2 interactome which could be useful for interpreting new findings in the context of the disease and could be further narrowed down by using expression or proteomic data. In our work, we used expression data from TISSUES 2.0 for sub-setting tissue-specific sub-networks and Gene Ontology to identify virus specific-proteins. This complete ACE2 network also provided a starting point for our new analysis, added to the manuscript of SARS-CoV-2 infected hiPSC-CMs, where those 68 genes served as seed nodes for the NERI algorithm which integrated co-expression networks with PPI networks. The workflow of the bioinformatic analyses performed in this study is shown in Figure 2.

**Figure 3:**
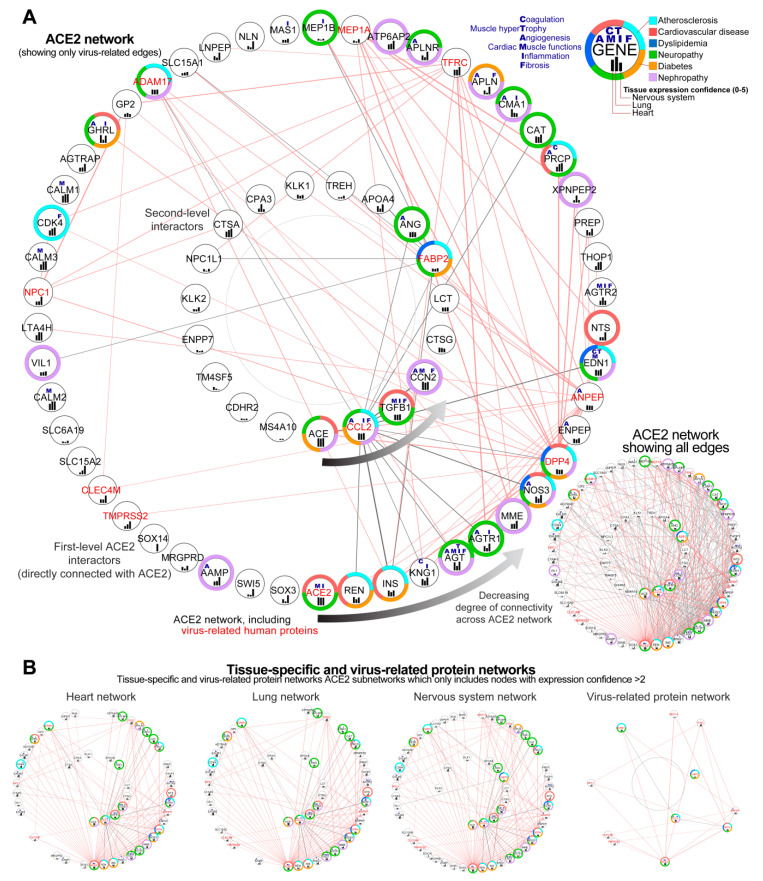
Predicted ACE2 (Angiotensin-Converting Enzyme 2) interaction network. (**A**) Complete ACE2 network visualized as two circles ordered by the number of connections (degree) with other nodes. A circular degree-sorted layout was used to enable us to hide the edges to simplify visualization. The external circle depicts the first level ACE2 interactors; the internal circle depicts the second level of interactors, with genes that do not connect directly with ACE2. For clarity, on the main figure, we showed only the edges associated with virus-related proteins (gene id in red). Edges associated with virus-related proteins are shown in red for first-level (direct) and in grey for the second-level (indirect) ACE2 interactors. Inset in the top right depicts the additional information for each gene/protein, as associated processes (blue letters), associated diseases (color-label ring), and expression confidence across key tissues (black bars). Inset in the bottom right depicts the same network including all edges. Genes present on the bottom, toward the right of the circular network, showed the highest connectivity within the network. Notice that the closest ACE2 interactors are ACE (Angiotensin-Converting Enzyme 1), renin (REN) and inulin (INS), which play a central role in the pathophysiology of a number of cardiovascular disorders. The following interactor is KNG1 (Kininogen 1), essential for blood coagulation and assembly of the kallikrein-kinin system and AGT (Angiotensinogen) influencing the renin-angiotensin system (RAS) function. In the network are present 11 virus-infection-related proteins (red labels) forming a dense connection with ACE2 and its top interactors which can affect its functionality. (**B**) Subsets of ACE2 network containing only highly expressed proteins in the heart, lung, and nervous system; analogous network for virus-related proteins (right). From the genes which did not have direct interactions with ACE2, the gene ACE showed the highest connectivity.

From the 68 genes included in the complete ACE2 network, only two did not show any interactions according to the String database which we used for the visualization of the network (Figure 3A). This figure depicts the complexity of this disease, presenting the possible primary alterations following SARS-CoV-2 infection, on level of proteins, and the secondary alterations on gene expression level (including initial ACE2 downregulation and later ACE2 overexpression). The genes from the ACE2 network were sorted using circular sorting by the number of connections with other genes to simplify the visualization

#### 3.2.1. Identification of Genes Showing the Highest Connectivity within the Complete ACE2 Network

Analysis of the complete interaction network between ACE2 and associated genes showed, as expected, the highest number of interactions between ACE2 and other genes (49 interactions), followed by ACE, which was not directly connected with ACE2 (33 interactions), renin (REN, 32 interactions), insulin (INS, 31 interactions), kininogen 1 (KNG1, 30 interactions) and angiotensinogen (AGT, 28 interactions) (Figure 3A).

#### 3.2.2. Identification of the Virus Infection-Related Proteins within the Complete ACE2 Network

Analysis of the ACE2 interaction network revealed 11 genes associated with virus infection-related ontological terms (ACE2, DPP4, ANPEP, CCL2, TFRC, MEP1A, ADAM17, FABP2, NPC1, CLEC4M, TMPRSS2) which could be especially affected in SARS-CoV-2 infection, leading to disturbance of the network. All these genes were connected directly with ACE2 according to the String database, except for gene FABP2 (six interactions with ACE2 neighbours) and CCL2 (15 interactions). From this group, the highest degree of connectivity with other genes from the network was found for DPP4 (22 interactions), ANPEP (19 interactions), and CCL2 (C-C Motif Chemokine Ligand 2) indirectly connected with ACE2 (15 interactions, as presented before). Two genes CDHR2 and MS4A10 did not have any known connections with other genes according to the String database, for edge confidence score cut-off > 0.4.

#### 3.2.3. Sub-Setting of the Tissues-Specific ACE-2 Related Networks

We also made subsets of complete ACE2 network to show interactions for heart tissue, lungs, and nervous system as well as virus-infection related proteins. Tissue-specific networks were selected based on their tissue expression confidence in analyzed tissues. These enabled us to evaluate similarities between selected tissues to predict the impact of ACE2 alterations in the heart tissue. Lung tissue was selected based on how affected it is by the SARS-CoV-2 infection and served as the control for our analysis. Nervous tissue was selected due to multiple neurological symptoms recently reported as associated with COVID19 disease [37].

We set this score as 2, which is relatively high taking into account scores for the genes from this database. For all tissues, the median expression confidence score was 0.914, for the heart 0.989, lung 1.319, and nervous system 1.556. We found 48 genes out of 68 overlapped between tissue-specific networks, and eight of them were virus-infection related proteins according to the GO database (Figure 3B). This ACE2-interactome also provided a starting point for our analysis of ACE2 co-expression networks in cardiomyocytes performed in this study, where those 68 genes served as seed nodes for the NERI algorithm which integrated co-expression networks with PPI networks.

Descriptions of the genes from the complete ACE2 network and link to its interactive version are available in Table S2.

### 3.3. Analysis of Changes in ACE2 Co-Expression Network in Infected Cardiomyocytes

To evaluate how alteration in the ACE2 network can affect cardiomyocytes we re-analyzed the GSE150392 GEO expression dataset for human-induced pluripotent stem cell-derived cardiomyocyte (hiPSC-CMs) 72 h post-infection with SARS-CoV2. We utilized the NERI algorithm [28,38] that integrates the PPI BioGrid interactome data with co-expression networks to take a closer look at the ACE2-related network in the infected cardiomyocytes. We used 68 genes from the predicted ACE2 network as seed genes, to focus on this region of the transcriptome. The goal of this analysis was to identify the genes and interactions between them that could be affected by the alterations in the ACE2 protein network caused by the virus and impact the functionality of cardiomyocytes. By assuming a network medicine hypothesis, the method explored the neighborhood of a gene set by locating paths possessing more co-expressed genes with seeds—this is independently performed for two conditions (control and disease). This approach enabled us to identify within a large co-expression network a central cluster of genes also called “hub nodes”, highly connected with other affected genes. Corroborating signals across affected hub nodes can result in changes in cellular signaling and/or transcript and protein expression levels of neighboring genes leading to pathological changes in cardiomyocytes, even when hub nodes themselves show little or no changes in expression. In order to identify those genes, we selected nodes and edges in which Rank numbers generated by NERI ranged from 1 to 200 in terms of the difference in co-expression between control and disease (Rank Delta) and changes in connectivity (Rank X).

#### 3.3.1. Identification of the ACE-2 Related Hub Genes Related to Corrupted Co-expression Networks

We identified 139 hub genes, four of them CAT (Catalase), AGT, AGTRAP, MME (reduced co-expression networks) and ATP6AP2 (enhanced co-expression networks) were also present among ACE2 seed genes. Decreased co-expression networks among top hub genes were observed for EGFR (Epidermal Growth Factor Receptor), FN1, TP53, FBXO6, RNF2, ELAVL1, PCNA, and HSP90AA1; while the strongest increase in co-expression network was observed for hub genes NTRK, COPS6, RAD51, PTEN, PSMA3, FRMD5, TRIM25 and APP.

#### 3.3.2. Identification of the Most Altered Connections between ACE2-Related Hub Genes

Most enhanced interactions were between HSP90AA1-MAST2 and ISYNA1 (ACE2 interactor) with TRIM25. The most diminished interactions were between APP-MAST2 and EGFR-MAST2. We also observed an enhancement of the co-expression network for EWS RNA Binding Protein 1 (EWSR1).

#### 3.3.3. Identification of the Most Altered Connections between ACE2-Related Seed Genes and Hub Genes

**Figure 4:**
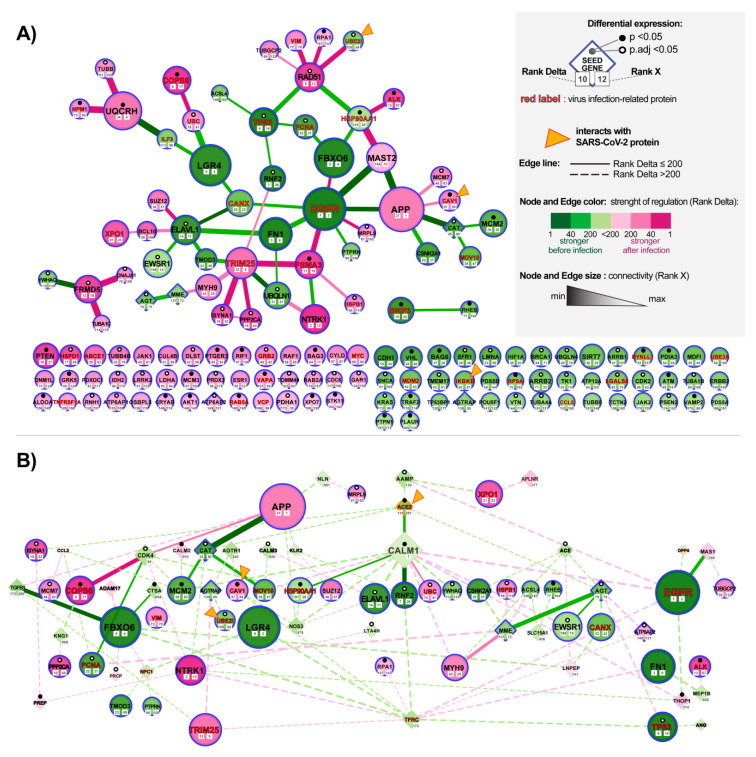
Alteration of ACE2 (Angiotensin-Converting Enzyme 2) networks in cardiomyocytes infected with SARS-CoV-2. (**A**) ACE-2 related hub genes with corroborated signaling obtained by analyzing expression data of human-induced pluripotent stem cell-derived cardiomyocytes (hiPSC-CMs) after 72 h of infection with SARS-CoV2. The network was constructed by using the NERI (Network by Relative Importance) algorithm which integrates protein-protein interaction (PPI) BioGrid interactome with gene co-expression network. For clarity, we selected top genes and edges which had Rank Delta and Rank S number between 1 and 200; additionally, we showed nodes with the best NERI scores (low-rank number) which did not have associated edges with best scores (low-rank number). Genes marked with orange triangles showed direct interaction with SARS-Cov-2 [39]. Notice that EGFR (Epidermal Growth Factor Receptor) and APP (Amyloid Beta Precursor Protein) showed the strongest alterations in their co-expression networks. (**B**) PPI network between top co-expressed hub genes from panel A (circular shapes) and seed genes (diamond shapes) related to the complete ACE2 network identified using data mining. The size of the nodes and weight of the edges is associated with Rank X number, related to biological importance, while color is associated with Rank Delta, related to the difference in co-expression network between control and disease. Notice that ACE2 shows a reduced number of connections, consistent with its initial downregulation in the early stage of infection; in later stages of infection, we can expect an inversion of observed regulation caused by the virus-related ACE2 overexpression [40].

**Figure 5:**
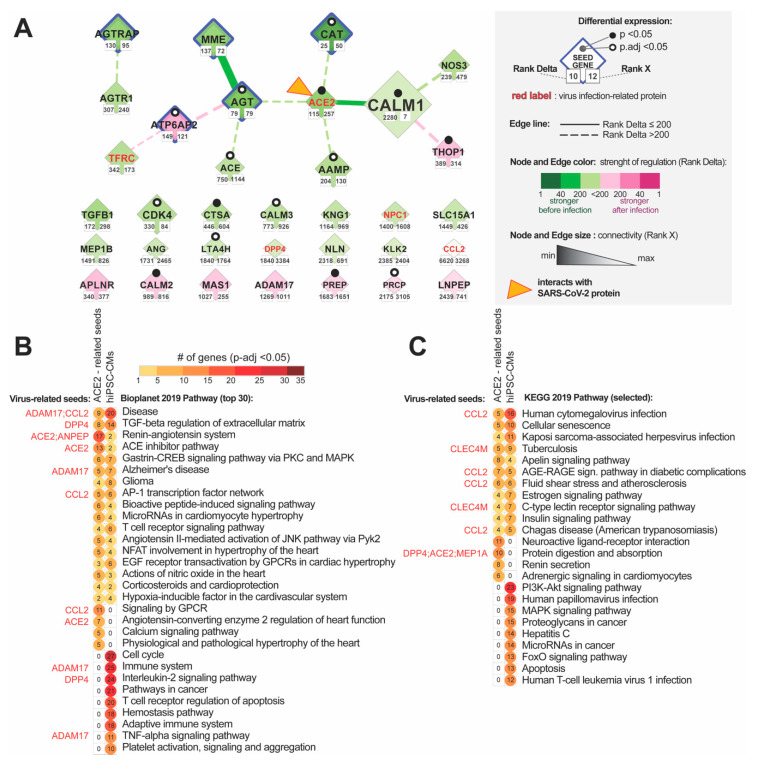
ACE2 (Angiotensin-Converting Enzyme 2) network in infected human-induced pluripotent stem cell-derived cardiomyocyte (hiPSC-CMs) and top signaling pathways enriched in ACE2 interaction networks. (**A**) ACE2 related genes identified using data mining and co-expression network in stem cell-derived cardiomyocytes (hiPSC-CMs) 72 h after infection. Pathway enrichment analysis of the complete ACE2 network (68 genes) and 139 top hub genes identified in ACE2 related co-expression network analysis for hiPSC-CMs. Notice that the strongest altered interaction was between AGT-MME (membrane metallo-endopeptidase) and ACE2 and CALM1 (Calmodulin 1), and the strongest affected nodes are AGT (Angiotensinogen) and CAT (Catalase). (**B**) Bioplanet database (top 30 pathways) and (**C**) KEGG database (additional pathways not present in Bioplanet). Virus-infection related proteins from the complete ACE2 network are marked with red font. All circles presented on the graph are associated with significantly enriched pathways (False Discovery Rate corrected *p*-value < 0.05). In this analysis, we also included ACE2-sub-networks for the heart, lungs, and nervous system, but due to high similarity with results for the complete ACE2 network, we excluded them from the figure for better clarity.

Analysis of interactions between the seed genes and hub genes showed the strongest alterations in the connection between CALM1 (Calmodulin 1) and RNF2 involved in cardiac development [9] (Figure 4B). Analysis of the alterations in connections between seed genes showed also affected ACE2-CALM1 interaction (RankDelta = 60, Rank X = 155) and AGT-MME (Rank Delta = 85, RankX = 63). ACE2 placed in terms of Rank Delta at 115, and Rank X placed 257 from 7844. In total, we identified 34 from our 69 seed genes showing changes in-expression networks. Fourteen of them were co-expressed with each other (Figure 5A)

#### 3.3.4. Identification of the Most Altered Connections between ACE2-Related Seed Genes

The strongest interaction between ACE2 and other genes was for seed gene CALM1 (Rank Delta = 2280, Rank X = 155). Generally, seeds were not expected to have high-Rank X numbers unless they were not a hub of interactions for other seed genes.

### 3.4. Enrichment Analysis of the Signalling within ACE2-Tissue-Specific Network

In order to compare our in silico predictions of the ACE2 network with its co-expression changes in infected cardiomyocytes, we performed enrichment analysis of signaling pathways using the EnrichR website (Figure 5). The aim also was to correlate those results with later disease predictions. We used 68 genes from the complete ACE-network and its subnetworks in the heart, lungs, and nervous system, as well as the top 139 hub genes identified by the NERI algorithm as most affected by the SARS-Cov-2 infection. Enrichment analysis of those networks showed multiple shared pathways associated with disease-related signaling, TGF-beta regulation of extracellular matrix, renin-angiotensin pathway, Alzheimer-disease, and AP-1 transcription factor network. Among top pathways shared between complete ACE2 networks, subnetworks and ACE2 network in cardiomyocytes, we observed many terms directly associated with heart functions, for example, microRNAs in cardiomyocyte hypertrophy, EGF receptor transactivation by G protein-coupled receptors (GPCRs) in cardiac hypertrophy, actions of nitric oxide in the heart, corticosteroids and cardio-protection. We also observed significant enrichment in all analyzed datasets of cellular senescence, apelin signaling, AGE-RAGE signaling pathway in diabetic complications, and estrogen signaling pathway identified using the KEGG database.

By comparing the complete ACE2 network to the co-expression in the hiPSC-CMs network, we observed in the former a higher number of genes associated with renin-angiotensin related signaling, ACE inhibitor pathway, renin secretion, protein digestion, and absorption. On the other hand, in infected cardiomyocytes, there are more genes related to cell cycle signaling, interleukin-2 signaling, cancer-related pathways, T cell receptor regulation of apoptosis, and hemostasis pathway. Additionally, the platelet-degranulation pathway was enriched in infected cardiomyocytes, but also in heart and lung subnetworks, but not in the complete ACE2 network.

### 3.5. Enrichment Analysis of the Disease Terms Associated with ACE2 Tissue-Specific Network

In order to identify the disease traits which would be helpful in precise identification of the risk groups of patients with COVID-19, we performed an enrichment analysis of the DisGenet disease and Rare Diseases AutoRIF database (associating genes with publications PubMed Ids) using the EnrichR website to evaluate phenotypes associated with ACE2 interaction in different tissues. This analysis guides the identification of phenotypes that can be triggered by ACE2-network alterations in selected tissues heart, lungs, nervous system, and hiPSC-CMs as well as virus-protein-related network and complete ACE2 network. Moreover, the analysis of rare traits, usually related to single genes enabled us to precisely characterize the consequences of alterations in the specific genes from the ACE2-network.

#### 3.5.1. Analysis of the ACE2-Related Common Diseases

**Figure 6:**
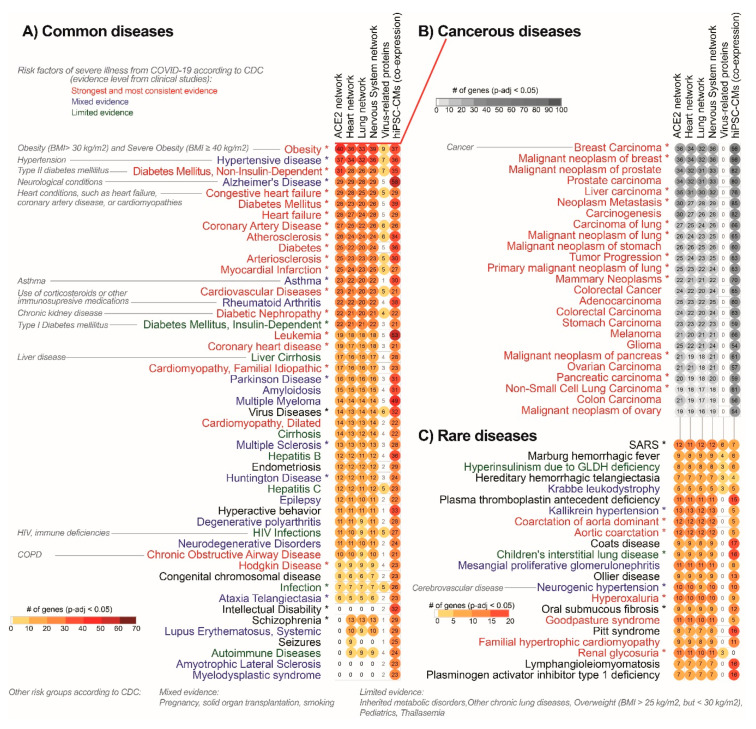
Top potential COVID-19 risk groups are significantly associated with ACE2 interaction networks. Risk groups are characterized as (**A**) common, (**B**) cancerous and (**C**) rare diseases. This list is based on enrichment analysis of a database of gene-disease associations (DisGeNET), analyzed through the EnrichR database. We performed disease enrichment analysis in the complete ACE2 network (leftmost column of symbols), and subsets of this network expressed in the heart, lung, and nervous system; we also performed this same analysis for 11 virus-infection related proteins (rightmost column) and ACE2-co-expression network in stem cell-derived cardiomyocytes (hiPSC-CMs) after 72 h of infection. Diseases marked with asterisks include the ACE2 gene. For heart, lung and nervous system tissue, we used the cutoff of expression confidence > 2, obtained from the Tissue2.0 database. All circles presented on the graph are associated with significantly enriched disease terms (False Discovery Rate corrected *p*-value < 0.05). Top cancer-related diseases are shown on panel B and were subset from the DisGeNET diseases list by using cancer-related keywords. Notice that top diseases are already known as major risk groups in COVID-19. Label colors are associated with evidence of underlying medical conditions that increase the risk of severe illness from COVID-19 according to the Centers for Disease Control and Prevention (CDC) [https://www.cdc.gov/coronavirus/2019-ncov/need-extra-precautions/evidence-table.html updated on 2 November 2020]. “Strongest and most consistent evidence” was defined as consistent evidence from multiple small studies or a strong association from a large study; “Mixed evidence” was defined as multiple studies that reached different conclusions about risk associated with a condition; and “Limited evidence” was defined as consistent evidence from a small number of studies. Unassigned terms are marked with black color.

The analysis of non-cancerous diseases in the DisGenet database revealed that the highest number of genes from all analyzed networks was associated with the following disease phenotypes (in decreasing order): numerous cancerous diseases, obesity, hypertensive disease, non-insulin-dependent DM, congestive HF, coronary artery disease and atherosclerosis, were observed in all analyzed networks (Figure 6A). Terms not enriched in the virus-related network but containing virus-infection related genes were: Alzheimer’s disease, heart failure, diabetes mellitus, asthma and rheumatoid arthritis. Cancer, Alzheimer’s disease and leukemia were the strongest enriched terms in infected cardiomyocytes.

#### 3.5.2. Analysis of ACE2-Related Cancerous Diseases

Cancerous diseases were grouped on the separate graph by using cancer-related key-words and showed the strongest enrichment of breast cancers and prostate-related cancers, as well as general carcinogenesis-related processes including neoplasm metastasis (Figure 6B).

#### 3.5.3. Analysis of ACE2-Related Rare Diseases

The analysis focused on rare diseases revealed that the most significant ones were SARS, blood-coagulation-related diseases (Marburg hemorrhagic fever, hereditary hemorrhagic telangiectasia, plasma thromboplastin deficiency, Coats disease), and multiple diseases associated with hypertension (Kallikrein hypertension and aortic coarctation) (Figure 6C). Among rare diseases, we also observed significant enrichment of Eclampsia (average 7.1 genes from each dataset), HELLP (haemolysis, elevated liver enzymes, low platelet count) syndrome (average 6.3 genes from each dataset) and Kawasaki disease (average 3.3 genes from each dataset) observed occasionally in COVID-19 [41].

### 3.6. Integration of ACE2 Network with SARS-CoV-2/Human Interactome

To identify how the ACE2 network is connected with the SARS-CoV-2/Human Interactome and how it could affect the heart, we combined the previously published SARS-CoV-2 interactome [39] with our complete ACE2 network and top findings from the co-expression network analysis in hiPSC-CMs 72 h after infection.

#### 3.6.1. Identification of ACE2-Related Genes Interacting with the Virus Proteins

We found that three proteins from our network, ACE2, CLEC4M, and TMPRSS2, directly interact with virus glycoprotein S. Top hub nodes identified in NERI analysis, CAV1 (Caveolin-1), UBE2I, and IKBKB, also showed connections with virus proteins ORF3a, N and M. In total, 45 proteins from the complete ACE2 network interact with 38 from 94 human host proteins for SARS-CoV-2.

#### 3.6.2. Identification of ACE2-Related Genes Interacting with the Host Proteins

**Figure 7:**
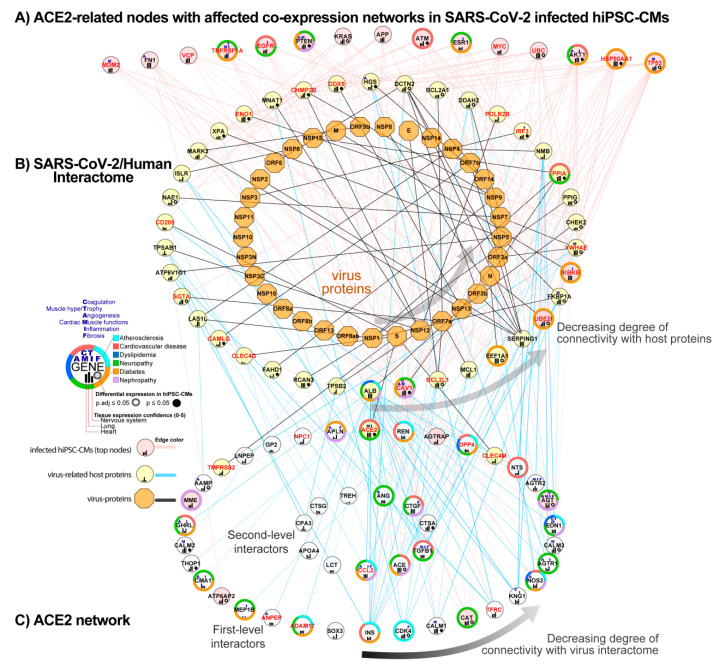
Combined ACE2 network with SARS-CoV-2/Human interactome and co-expression network in infected human-induced pluripotent stem cell-derived cardiomyocytes (hiPSC-CMs). (**A**) Hub nodes identified in the co-expression network analysis of infected hiPSC-CMs using the NERI algorithm, showing the highest connectivity with SARS-CoV-2/Human interactome (top 15 genes). (**B**) SARS-CoV-2/Human interactome as shown in previously published work [39]. (**C**) ACE2 network components that interact with SARS-CoV-2/Human interactome proteins. Nodes from network A which have the highest connectivity are sorted from right to left. Nodes from the networks B and C are circularly sorted by the number of connections with virus interactome. Virus proteins are shown as orange octagons, while virus-infection related human proteins have red labels.

We also found that SARS-CoV-2 interactome strongly connects with 23 hub genes which were also the strongest affected in hiPSC-CMs co-expression network analysis including EGFR, APP, FN1, and TP53. SARS-CoV-2 interactome strongly connects with the complete ACE2 network through INS, CDK4 (Cyclin-Dependent Kinase 4), CCL2, and ALB (Albumin), all of them associated with atherosclerosis processes. The strongest connection between the complete ACE2 network and SARS-CoV-2/Human Interactome was with INS, which interacted with 12 host proteins from SARS-CoV-2/Human Interactome. Other top interactors were seed genes CAT, CCL2, CDK4, CALM1 connected with at least six host proteins. The strongest interaction for ACE2-related co-expression networks in hiPSC-CMs was between HSP90AA1 and TP53, connected with 16 host proteins from the SARS interactome. Among host proteins directly interacting with virus proteins, the strongest connection with the ACE2 network occurs by ALB (30 interactors form ACE2 network) and CAV1 (13 interactors) which had affected the co-expression network after virus infection in hiPSC-CMs (Figure 7).

### 3.7. ACE2 Network-Related miRNA Predictions

**Figure 8:**
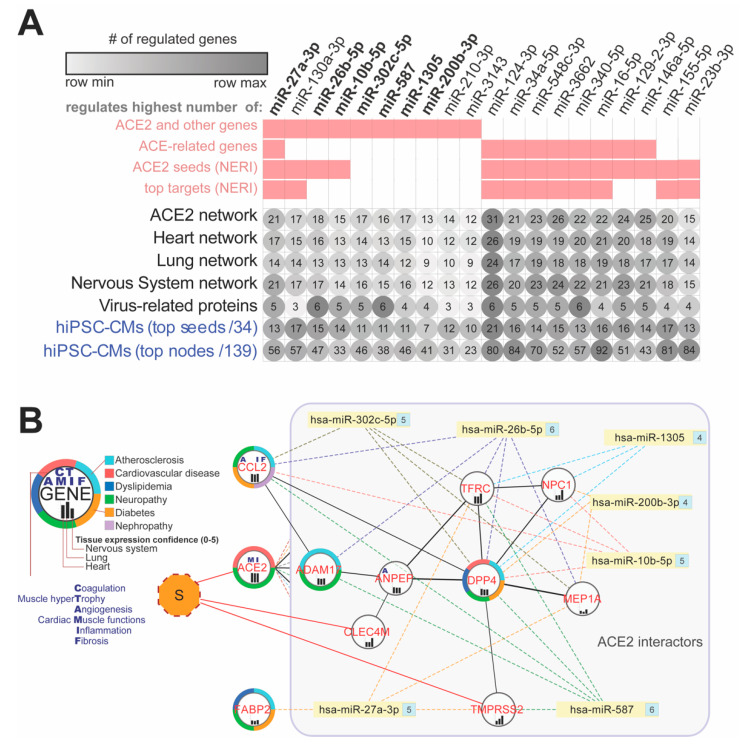
Top 20 potential miRNA modulators of the ACE2 network in COVID-19. (**A**) Top miRNAs regulating the highest number of genes within all ACE2 related-networks. Pink squares are showing if miRNA was present among the top 10 miRNAs in a given dataset. (**B**) Interaction network between virus-infection related proteins (red labels) and top miRNAs (bolded label on the A panel) regulating ACE2 and shared between analyzed networks and regulating at least four virus-related proteins from the complete network. Numbers on the right side of the miRNAs depict the number of targeted genes within the network. CCL2 and FABP2 genes are not direct interactors of the ACE2, so they are presented outside of the ACE2-interactors box. “S” refers to SARS-CoV-2 spike glycoprotein S. Human-induced pluripotent stem cell-derived cardiomyocytes (hiPSC-CMs).

In order to identify miRNAs which could play a regulatory role in COVID19, and especially its cardiovascular consequences, we performed miRNA-target predictions using the following sets of genes: 69 genes from the complete ACE2 network, gene lists from its subnetworks for heart, lungs, nervous system, and virus-related proteins, as well as top hub genes from the ACE2 co-expression network in hiPSC-CMs. Because among the top nodes identified in the co-expression analysis of hiPSC-CMs only four seed genes were present, we decided to also include for target predictions ACE-related seed genes which showed interactions with the top hub genes. For improving the precision of predictions, we analyzed miRNAs that showed expression in blood, serum, or plasma according to the TISSUES 2.0 database. In further analyses, we focused on miRNAs which regulated the highest number of the genes from the network as well as including ACE2. Analysis of the top 10 miRNAs regulating each from those seven networks revealed overall 10 miRNAs, also regulating the ACE2 gene (Figure 8A). Seven (hsa-miR-302c-5p, hsa-miR-27a-3p, hsa-miR-1305, hsa-miR-587, hsa-miR-26b-5p, hsa-miR-10b-5p, hsa-miR-200b-3p) were also regulating a high number of virus-related proteins (Figure 8B). Among miRNAs shared across analyzed networks that were not regulating ACE2 but were present among the top 10 in all analyzed datasets, we identified i.e., hsa-miR-124-3p, hsa-miR-34a-5p, hsa-miR-548c-3p and hsa-miR-16-5p.

## 4. Discussion

In the current study, we characterized the interaction between SARS-CoV-2 infection and ACE2 functional networks with a focus on CVD. Using an integrative multi-omic approach, we described the ACE2 interaction network and evaluated its expression using the hiPSC-CMs dataset. The approach of integrating multiple sources of biological data used in our study is becoming increasingly popular [42]. There are currently numerous databases integrating and systematizing data from different levels of biological regulation making this knowledge easily accessible. To improve the identification and prioritization of genes associated with complex diseases, some works have begun to integrate PPI networks with information derived from other omics data, which have contributed to a better understanding of gene functions, interactions, and pathways [25,43,44]. The integration of PPI networks and gene expression data has also improved disease classification and identification of disease-specific deregulated pathways in COVID19 [45,46,47].

The main findings of our analysis are the following: (1) Expression of ACE2 is similar in lungs and heart, which provides a rationale why the cardiovascular system is also a target of SARS-CoV-2 infection; (2) Analysis of co-expression networks in infected hiPSC-CMs identified multiple hub genes, highly connected with other affected genes and associated with cardiovascular risk factors; (3) SARS-CoV-2 binding to the ACE2 receptor leads to major disturbances in signaling pathways linked to cardiac adverse outcomes in COVID19; (4) Analysis of ACE2 interaction networks revealed its association with numerous diseases including main COVID19 co-morbidities; (5) Analysis of the SARS-CoV-2 interactome revealed extensive connections with the top regulators of the ACE2 network; (6) We identified multiple miRNAs regulating ACE2 network (hsa-miR-302c-5p, hsa-miR-27a-3p, hsa-miR-1305, hsa-miR-587, hsa-miR-26b-5p, hsa-miR-10b-5p, hsa-miR-200b-3p; hsa-miR-124-3p and hsa-miR-16-5p).

Our hypothesis states that major implications of COVID19 cardiovascular outcomes are related to alteration in ACE2 receptor signaling associated with SARS-CoV-2 binding. Due to the difference between tissue-specific ACE2 networks, different pathological processes can be triggered in different organs. Our comparative analysis of multiple tissues showed consistency in crucial pathways including RAS signaling. This could help explain cardiac abnormalities presented in COVID-19 patients, including elevated troponin, myocarditis, arrhythmias, and sudden cardiac death [48]. Moreover, as the myocardial interaction network of ACE2 and its own expression is altered in patients with coexisting CVD, SARS-CoV-2 infection may result in greater damage to cardiomyocytes and account for greater disease acuity and poorer survival. This is consistent with recent data regarding the analysis of RNA-seq data of SARS-Cov-2 infected patients alongside controls: ACE2 network alteration seems to be the source of most recognized manifestations of COVID19 disease [40].

### 4.1. The High Expression of ACE2 in the Cardiovascular System Explains Why SARS-CoV-2 Infection May Target the Heart

The analysis focusing on the ACE2 tissue-specific expression showed that lungs and respiratory systems have similar ACE2 and TMPRSS2 expressions as the heart and cardiovascular systems and the nervous system. Recent publications regarding the impact of SARS-CoV-2 on the heart tissue showed that cardiomyocytes can indeed be infected with the virus [13,14].

### 4.2. The High Expression of ACE2 in the Reproductive System and Endocrine Glands and Enrichment of Cancerous Diseases Related to Those Organs Suggest Wider Implications of SARS-CoV-2

In our analyses, we also observed strong confidence in ACE2 expression in urogenital and endocrine tissues. Moreover, we found that the highest expression of TMPRSS2 gene which is known to positively regulate viral entry into the host cell via proteolytic cleavage of ACE2 is present in in the genitourinary tracts. There are studies supporting the high expression of ACE2 in the urogenital tissues [49,50,51] and endocrine tissues [52]. Additionally, there is increasing evidence of symptoms like male infertility [53,54], urinary tract inflammation linked to viral cystitis [55], and transmission of the virus through urine [56]. On the other hand, so far qPCR experiments to detect SARS-CoV-2 in semen and testicular biopsy did not find signs of virus infection [50]. We hypothesize that in this case disturbances in urogenital tissues could be associated with alternative regulation of the ACE2 network. Additionally, our analyses using the NERI algorithm showed strong alterations in cancer-related TP53, NTRK, COPS6, and RAD51 co-expression networks and exceptionally high enrichment of cancerous processes associated with reproductive organs. Thus, we predict that additional impacts of SARS-CoV-2 infection can be observed much later than acute infection. Interestingly, TP53 is considered a master regulator of maintenance of cardiac tissue transcriptomic homeostasis [57].

### 4.3. ACE, REN, INS, KNG1, and AGT Play A Role in Cardiovascular Risk Factors Related to SARS-CoV-2 Binding to the ACE2 Receptor

In our study ACE, REN, INS, KNG1, and AGT showed the highest connectivity within the complete ACE2 network and association with enriched diseases that are known to be a risk factor for a severe course of COVID-19. Our analysis of the data related to infected hiPSC-CMs confirmed changes in co-expression networks of ACE, AGT (also present among hub genes), and KNG1. Both ACE and AGT also showed differential expressions in hiPSC-CMs. On the other hand, expression levels of INS and REN were extremely low thus suggesting rather their role other than in cardiac tissues. ACE and ACE2 interplay with the RAS pathway and plasma kallikrein-kinin system (KKS), of which a component is KNG1, a hormonal pathway that modulates the intrinsic blood coagulation system, angiogenesis, the complement pathway and bradykinin-related inflammation. Deregulation of KKS may result in thromboembolic complications and lead to sepsis exacerbation in infections [58,59,60]. Deregulation of AGT which is a crucial component of the RAS pathway is associated with the pathogenesis of essential hypertension and atrial fibrillation.

### 4.4. Analysis of Infected hiPSC-CMS Using the NERI Algorithm Revealed Affected Co-expression Networks of EGFR, APP, and CALM1 Implicating Their Role in Cardiac and Thromboembolic Complications

EGFR loss in vascular smooth muscle cells and cardiomyocytes leads to arterial hypotension and cardiac hypertrophy [61]. EGFR was found as a pivotal regulator of thrombin-mediated inflammation. It also plays a role in changes from lethal to non-lethal influenza infections [56,62]. EGFR showed decreased signaling in infected hiPSC-CMs.

APP is a precursor protein for Amyloid-beta (Aβ) peptide which is massively released from the blood to nearby tissue upon the activation of platelets and has strong antibiotic activity against viruses, bacteria, and fungi [63]. Accumulation of Aβ in tissues is observed in Alzheimer’s disease, glaucoma, cancerous disease, myocardium with diastolic dysfunction, and the placenta during preeclampsia [64,65]. All those diseases were enriched in our study. APP showed an increased co-expression network in infected hiPSC-CMs. Identification of this interplay between EGFR and APP can play an important role in intervention targets in COVID19 treatment.

The analysis focusing on the close ACE2 interactors revealed its strongly affected connection with CALM1 in infected hiPSC-CMs. CALM1 regulates the function of ion channels playing a role in platelet aggregation and cardiomyocytes activity. It is also an important ACE2 interactor playing a role in viral pathogenesis [66,67]. In our analysis, CALM1 showed interactions also with hub node PTEN affecting cardiomyocyte contractions and growth.

### 4.5. Alterations in the ACE2 Interaction Network Can Aggravate Major Comorbidities in COVID19 through Related Signalling Pathways

Our analysis of ACE2 interaction networks including co-expression networks in infected cardiomyocytes showed that change in ACE2 receptor activity can lead to significant disturbances in signaling pathways linked to well-known complications in COVID-19. These pathways included TGF-beta regulation of extracellular matrix, renin-angiotensin system AP-1 transcription factor network, apelin signaling, AGE-RAGE signaling pathway in diabetic complications, and estrogen signaling. Signaling pathways most affected in hiPSC-CMs were related to cell cycle (i.e., pathways in cancer, PI3K-Akt signaling pathway), immune system (i.e., interleukin-2 signaling pathway, T-cell receptor regulation of apoptosis), hemostasis and platelet activation. Those results were supported by our subsequent analysis of disease-related phenotypes which are the major comorbidities in COVID19 cancerous diseases, obesity, hypertensive disease, diabetes, and Alzheimer’s disease.

### 4.6. Renin-Angiotensin Pathway, AGE-RAGE, and Apelin Signalling as Fundamental Mediators of the Blood Pressure Dysregulation Mediated through ACE2 in COVID-19

In our in silico analysis using ACE2 functional networks, we found that RAS/ACE2, AGE-RAGE, and Apelin signaling pathways play an important role in SARS-CoV-2 infection. These pathways have a crucial role in the pathogenesis of DM, CVD, and blood pressure regulations. Abnormalities of ACE2/RAS pathway signaling and deregulation of angiotensin II as a fundamental mediator of this axis are closely related to the pathophysiology of hypertension and progression of cardiovascular remodeling [68,69]. Therefore, binding of the SARS-CoV-2 to the ACE2 receptor leading to disturbances in the pathways of these key regulators might explain the adverse outcome in COVID-19 patients with the coexistence of the above mentioned clinical conditions. Apelin signaling is also involved in many physiological processes such as energy metabolism, blood pressure regulation, and cardiac contractility and plays an important role in organ and tissue pathologies including DM, obesity, HF, and HIV-1 infection [70]. After the virus enters the cells, ACE2 is likely to decrease its activity, thus favoring an increase of the ACE/ACE2 balance toward the prevalence of the ACE arm in the RAS which causes an increase of ROS production, vasoconstriction, and inflammation [71]. On the other hand, a recent COVID-19 related study showed decreased expression of ACE in combination with increases in ACE2 likely causing bradykinin-related increases in vascular dilation, vascular permeability and hypotension, explaining many of the symptoms being observed in COVID-19 [40].

### 4.7. The Role of Virus-Infection Related Proteins from ACE2 Network in COVID-19 Adverse Outcomes

Analysis of the connection between SARS-CoV-2 interactome revealed direct interaction of the virus glycoprotein S with 3 of the 11 virus-infection related proteins identified as incomplete in the ACE2 network (ACE2, CLEC4M, and TMPRSS2). We also identified DE hub nodes (CAV1, UBE2I) interacting with other virus proteins. Interestingly, CAV1 was identified as a possible alternative receptor for SARS-CoV and Canine respiratory coronavirus (CRCoV), which may be associated with the virus infection, replication, assembly, and budding [72,73]. CAV1 showed an enhanced co-expression network. Additionally, we observed multiple connections between host genes from virus interactome and most affected genes in hiPSC-CMs. Those genes included TP53, HSP90AA1 but also ESR1, FN1, APP and EGFR and seed genes CAT, AGT, AGTRAP, DPP, CCL2 and MME. The especially interesting genes are discussed below.

HSP90AA1 was recently shown in pre-print as reducing SARS-CoV- 2 viral replication and TNF and IL1B mRNA levels [74]. Additionally, in our analysis, the strongest affected interaction in cardiomyocytes was observed between HSP90AA1 and MAST2. MAST2 regulates IL12 production in macrophages and shows association with red blood cell distribution width which was identified recently as a biomarker of COVID19 mortality [75]. Interestingly, MAST2 also showed an affected connection with the top hub gene APP. HSP90AA1 and seed gene CAT showed significant differential expression and a strong reduction in co-expression networks in infected hiPSC-CMs in our study. CAT is considered the most effective catalyst for the decomposition of H2O2, regulating the production of cytokines, protecting from oxidative injury, and repressing replication of SARS-CoV-2 [76].

FN1 in our study showed decreased signaling in hiPSC-CMs. FN1 inhibition attenuates fibrosis and improves cardiac function in a model of heart failure [77]. Interaction of human plasma fibronectin with viral proteins of HIV suggests that its binding to virus particles may reduce viremia and thus may be involved in the clearance of viral proteins from the cells [78].

Higher plasma DPP4 (Dipeptidyl Peptidase 4) can be found among patients with obesity, metabolic syndrome, and DM, who are at risk of a severe course of COVID-19 [79]. DPP4 knock-in mice were found more susceptible to MERS-CoV infections which resulted in the severe inflammatory response and lethal lung disease [80,81]. Therefore, it should be further investigated whether DPP4 inhibitors, widely used for the treatment of DM, may act as therapeutic drugs for ARDS caused by SARS-CoV-2 infection [82]. Our study revealed the possible interaction between DPP4 and EGFR which was identified as the most affected gene in co-expression network analysis.

Among affected virus-related-infection proteins, we also identified CCL2. CCL2 protein has been implicated in lung inflammatory disorders and contributes to the development of pulmonary fibrosis [23,83]. It is worth mentioning that among SARS-CoV-infected patients the level of pro-inflammatory cytokines, especially CCL2 and TGF-β1 (both affected in infected hiPSC-CMs) were increased in cells expressing ACE2, while this could not be seen in tissue with undetectable ACE2 expression [84]. A comparable pattern of inflammatory cytokines was also found in SARS-CoV-2 infection [85]. This makes CCL2 a promising link between ACE2 and cytokine storm associated with severe COVID-19 disease.

Altogether, our results suggest that not only is ACE2 affected by the entrance of the virus to the cardiomyocytes, but that this virus also affects multiple ACE-2 interactors and that the ACE-2 network can also be a part of the virus propagation machinery.

### 4.8. miRNAs as Promising Antiviral Modulators of the ACE2 Network and a Potential Biomarker of HF Associated with COVID-19

In the initial step of our study, we identified miRNAs that may regulate the expression of ACE2 networks and related processes. To our best knowledge, we present here novel results on the potential role of miRNAs as a diagnostic and prognostic tool in heart muscle injury in the course of SARS-CoV-2 infection

#### 4.8.1. miR-1305 and miR-587: TGF-β Signaling Pathway Regulators in HF Progression

MiR-1305 and miR-587 were found to regulate the expression of TGF-β pathway members related to virus infections and lymphocytes T activation, Mothers Against DPP Family Members - SMAD3, and SMAD4) [86,87,88,89], ventricular remodeling, myocardial fibrosis and hypertrophy and, as a result, HF progression [90]. The highest expression of miR-587 was found in platelets of patients with acute coronary syndrome and was closely related to the severity of coronary artery stenosis [91].

#### 4.8.2. miR-26b-5p: Anti-fibrotic Agent and AGTR1-Dependent Hypertension Modulator

In our study, we found that miR-26b-5p may play an important role in the pathogenesis of HF in COVID-19 patients. Noteworthy, literature data suggest that AGTR1 (Angiotensin II Receptor Type 1) can modulate hypertension, via the regulation of miR-26b-5p in arachidonic acid metabolism. Additionally, miR-26b-5p has an anti-fibrotic effect in the liver, in the diabetic mouse myocardium, and Ang-II-induced mouse cardiac fibroblasts [92].

#### 4.8.3. miR-302c-5p: Potential Antiviral Therapeutic and Biomarker of HF

Another miRNA from our network affecting ACE2 was miR-302c-5p, playing an important role in many viral infections [93,94,95]. A study reported an association between the miR-302 and cytokine storm and showed the potential of miR-302 as an antiviral therapeutic [95]. Our bioinformatic analysis for the first time showed the importance of miR-302c-5p in SARS-CoV-2 infection. Apart from the crucial function of miR-302 in viral infections, it may be also associated with CVD [96,97], as circulating miR-302 was positively correlated with NT-proBNP levels in acute HF patients and showed strong potential as a novel biomarker for the diagnosis and the differentiation of disease severity of acute HF [96].

#### 4.8.4. miR-27a-3p: A Potential Biomarker of Acute HF and NF-κB Signaling Regulator

miR-27a-3p targeting ACE2 and other genes from its network in our study were also found to be involved in the inflammatory response and oxidative stress through several pathways including PPAR-γ, NF-κB, and PI3K/AKT/Nrf2 signaling [98,99,100]. In the animal model of acute lung injury, expression of miR-27a-3p in alveolar macrophages was significantly decreased, while overexpression of miR-27a-3p suppressed NF-κB activation and alleviated acute lung injury by binding to its target NFKB1 [98]. Moreover, it was also found that miR-27a-3p may target pathways related to arthero-sclerosis and may act as a potential biomarker of acute HF [101,102].

#### 4.8.5. Hsa-miR-16-5p: Modulates Inflammatory Signaling and Cytokines including IL-1β, IL-6, and TNF-α, NF-κB mTOR-Related Pathways

hsa-miR-16-5p was found to affect a phenotypic change of T cells, modulate inflammatory signaling and cytokines including IL-1β, IL-6 and TNF-α, NF-κB mTOR-related pathways and genes [103,104]. Additionally, MiR-16-5p has been linked with the pathogenesis of several infectious diseases such as HIV-1 infection and malaria [105,106,107]. It is worth mentioning that miR-16-5p as a plasma diagnostic biomarker is able to distinguish severe and mild viral infections [108] and early HIV-1 infection from healthy individuals [106,109].

#### 4.8.6. Hsa-miR-124-3p: Has a Potentially Aggravating Role in Cardiovascular Consequences of COVID19

MiR-124-3p was identified as regulating the highest number of genes in hiPSC-CMs. The literature data show its aggravating role in failing hearts by suppressing CD151-facilitated angiogenesis [110]. miR-124-3p dysregulates NSC maintenance through repression of the transferrin receptor (TFRC—Transferrin Receptor) in Zika virus infection [111]. TFRC is linked with cardiomyopathy and was identified in our study as closely interacting with ACE2 in infected cardiomyocytes and is strongly connected with SARS interactome.

## 5. Conclusions

This comprehensive analysis provides novel information regarding the complexity of signaling pathways of SARS-CoV-2 infection affecting the cardiovascular system with a focus on cardiomyocytes, and forms a basis for the creation of predictive tools and introduction of therapy to improve outcome in COVID-19, and therefore has a potential to reduce economic consequences of the global pandemic. We believe that the results of our analysis could be further validated in laboratory and clinical settings and help to create a paradigm for future studies in this field. MiRNAs identified for the first time in this study can serve as potential biomarkers helping with the identification of the pathological changes in COVID-19 or serve as therapeutic targets, due to their stability in the serum, forming a basis for personalized therapy.

## Supplementary Materials

The website will be completed after article number is confirmed https://www.mdpi.com/2077-0383/9/11/3743/s1; Table S1. List of GO terms used for annotation of the genes to specific processes and functions; Table S2. Description of genes associated with complete ACE2 interaction network

## Abbreviations

ACE2	Angiotensin-Converting Enzyme 2
ACE	Angiotensin-Converting Enzyme 1
ARDS	Acute respiratory distress syndrome
COVID-19	Coronavirus disease 2019
CVD	Cardiovascular disease
DEG	Differentially expressed genes
DM	Diabetes mellitus
eNOS	Endothelial nitric oxide synthase
GEO	Gene Expression Omnibus
GO	Gene Ontology
GPCRs	G-protein-coupled receptors
GTEx	Genotype-Tissue Expression
HF	Heart failure
HK	High molecular weight kininogen
hiPSC-CMs	Human-induced pluripotent stem cell-derived cardiomyocytes
INS	Insulin
KKS	Kallikrein-Kinin system
KNG1	Kininogen 1
MERS	Middle-East respiratory syndrome coronavirus
MI	Myocardial infarction
miRNAs, miR	MicroRNAs
NERI	NEtwork by Relative Importance
NT-proBNP	N-terminal pro-B-type natriuretic peptide
PPI	Protein–protein interaction
RAS	Renin–angiotensin system
REN	Renin
ROS	Reactive oxygen species
SARS-CoV-2	Severe acute respiratory syndrome coronavirus 2
TMPRSS2	Transmembrane protease serine 2
Gene/protein	
ACE2	Angiotensin-Converting Enzyme 2
ADAM17	ADAM metallopeptidase domain 17
AGT	Angiotensinogen
AGTR1	Angiotensin II Receptor Type 1
ALB	Albumin
APP	Amyloid Beta Precursor Protein
ANPEP	Alanyl Aminopeptidase
APN/CD13	Aminopeptidase N/CD13
ATP6AP2	ATPase H+ Transporting Accessory Protein 2
CALM1	Calmodulin 1
CAT	Catalase
CAV1	Caveolin-1
CCL2	C-C Motif Chemokine Ligand 2
CCN2	Cellular Communication Network Factor 2
CDHR2	Cadherin Related Family Member 2
CDK4	Cyclin-Dependent Kinase 4
CLEC4M	C-Type Lectin Domain Family 4 Member M
CTSA	Cathepsin A
CTSG	Cathepsin G
DPP4	Dipeptidyl Peptidase 4
EGFR	Epidermal Growth Factor Receptor
ENPEP	Glutamyl Aminopeptidase
EV71	Enterovirus 71
EWSR1	EWS RNA Binding Protein 1 (EWSR1)
FABP2	Fatty acid-binding protein 2
FN1	Fibronectin 1
FoxO2	Forkhead box O-2
HSP90AA1	Heat Shock Protein 90 Alpha Family Class A Member 1
INS	Insulin
KNG1	Kininogen 1
KPNA2	Karyopherin Subunit Alpha 2
LNPEP	Leucyl And Cystinyl Aminopeptidase
LTA4H	Leukotriene A4 hydrolase
MAPK	Mitogen-activated protein kinase
MAST2	Microtubule Associated Serine/Threonine Kinase 2
MCP-1	Monocyte chemoattractant protein-1
MEP1A	Meprin A subunit alpha
MME	Membrane metallo-endopeptidase
MS4A10	Membrane Spanning 4-Domains A10
NFKB1	Nuclear Factor Kappa B Subunit 1
NF-κB	Nuclear factor kappa-light-chain-enhancer of activated B cells
NPC1	Niemann-Pick disease, type C1
PAI-1	Plasminogen activator inhibitor-1
PDGFR-b	Platelet-derived growth factor receptor-beta
PPARγ	Peroxisome proliferator-activated receptor-gamma
PRCP	Prolyl-carboxypeptidase
TFRC	Transferrin Receptor
TGFB1	Transforming Growth Factor Beta 1
THOP1	Thimet Oligo-peptidase 1
TMPRSS2	Transmembrane protease, serine 2
VEGFR2	Vascular endothelial growth factor receptor 2
**Signaling pathways**	
AGE-RAGE	Advanced glycation end products-Receptor for Advanced glycation end products
ERK1/2/AP-1	Extracellular signal-regulated kinases 1/2/AP-1
PI3K/AKT/Nrf2	Phosphatidylinositol 3′-kinase/AKT/NF-E2-related factor 2
ERK1/2/AP-1	Extracellular signal-regulated kinases 1/2/AP-1
PI3K/AKT/Nrf2	Phosphatidylinositol 3′-kinase/AKT/NF-E2-related factor 2
RAS	Renin-Angiotensin System
